# “The wrong tools for the right job”: a critical meta-analysis of traditional tests to assess behavioural impacts of maternal separation

**DOI:** 10.1007/s00213-022-06275-6

**Published:** 2022-11-23

**Authors:** Olivia Stupart, Trevor W. Robbins, Jeffrey W. Dalley

**Affiliations:** 1 Department of Psychology, University of Cambridge, Downing St, Cambridge, CB2 3EB UK; 2 Department of Psychiatry, Hershel Smith Building for Brain and Mind Sciences, Cambridge, CB2 OSZ UK; 3 Behavioural and Clinical Neuroscience Institute, University of Cambridge, Downing St, Cambridge, CB2 3EB UK

**Keywords:** Early life adversity, Stress-related disorders, Elevated plus maze, Sucrose preference test, Forced swim test, Endophenotypes

## Abstract

**Rationale:**

Unconditioned tasks in rodents have been the mainstay of behavioural assessment for decades, but their validity and sensitivity to detect the behavioural consequences of early life stress (ELS) remains contentious and highly variable.

**Objectives:**

In the present study, we carried out a meta-analysis to investigate whether persistent behavioural effects, as assessed using unconditioned procedures in rats, are a *reliable* consequence of early repeated maternal separation, a commonly used procedure in rodents to study ELS.

**Methods:**

A literature search identified 100 studies involving maternally separated rats and the following unconditioned procedures: the elevated plus maze (EPM); open field test (OFT); sucrose preference test (SPT) and forced swim task (FST). Studies were included for analysis if the separation of offspring from the dam was at least 60 min every day during the pre-weaning period prior to the start of adolescence.

**Results:**

Our findings show that unconditioned tasks are generally poor at consistently demonstrating differences between control and separated groups with pooled effect sizes that were either small or non-existent (EPM: Hedge’s g =  − 0.35, *p* = 0.01, OFT: Hedge’s g =  − 0.32, *p* = 0.05, SPT: Hedge’s g =  − 0.33, *p* = 0.21, FST: Hedge’s g = 0.99, *p* = 0.0001). Despite considerable procedural variability between studies, heterogeneity statistics were low; indicating the lack of standardization in the maternal separation protocol was the not the cause of these inconsistent effects.

**Conclusions:**

Our findings indicate that in general, unconditioned tests of depression and anxiety are not sufficient to reveal the full behavioural repertoire of maternal separation stress should not be relied upon in isolation. We argue that more objective tasks that sensitively detect specific cognitive processes are better suited for translational research on stress-related disorders such as depression.

**Supplementary Information:**

The online version contains supplementary material available at 10.1007/s00213-022-06275-6.

## Introduction

The World Health Organisation (WHO) estimates that at least 5% of all adults presently suffer with depression, now the leading cause of morbidity worldwide (WHO 2022). However, despite this burden, advances in our understanding of the aetiology and treatment of depression have slowed since the introduction of selective serotonin reuptake inhibitors or SSRIs (Hyman 2013; Malhi and Mann 2018). This unmet need extends to the deleterious effects of early life stress (ELS), a widely recognised risk factor for depression and anxiety disorders (Chandan et al. 2019; Hughes et al. 2017; WHO 2020). Indeed, roughly one in five adults in the UK report having suffered from neglect, physical, sexual, or emotional abuse during childhood, with roughly half of these individuals reporting several forms of abuse (Elkin 2020). Child abuse, along with other forms of childhood trauma and uncontrollable stress, including the loss of a parent, a parent with a mental illness or witnessing domestic violence, collectively make up Adverse Childhood Experiences (ACEs) — a commonly used term to describe a variety of early life stresses (ELS) (CDC 2021; Hughes et al. 2017). ELS exposure notably increases the incidence of morbidities later in life, specifically mental health disorders such as depression and anxiety (Chandan et al. 2019; Hughes et al. 2017).

Translational experimental approaches are widely used to investigate the mechanisms underlying symptom-based diagnoses in humans, which extend to the effects of ELS on the neurobiology of depression and other mental health disorders. However, humans exposed to ELS do not always develop depression later in life (Hughes et al. 2017) and there are no empirical biomarkers of depression at present (de Aguiar Neto and Rosa 2019; Gururajan et al. 2016; Lopez et al. 2018). Experimental approaches in animals are therefore necessary for gaining a deeper mechanistic understanding of ELS that would inform novel therapeutic intervention. Repeated maternal separation (MS) is a form of ELS that involves the separation of young animals from their mother for at least 60 min each day for several days during the pre-adolescent period (Schmidt et al. 2011; Tractenberg et al. 2016). MS produces long lasting immunological (Dutcher et al. 2020), neurobiological (Nishi 2020) and behavioural (Nishi 2020) outcomes in animals that resemble the effects of early life adversity in humans (Heim et al. 2008). However, the specific behavioural changes pertaining to depression-relevant phenotypes in MS animals have been difficult to identify because behavioural validation is often assessed using "quick and dirty" tests derived from observations of spontaneous reactions to various stimuli or contexts (Reardon 2019). As examples, the elevated plus maze (EPM) and open field test (OFT) both exploit a rodent’s intrinsic fear of open spaces (Treit et al. 1993), such that when animals are more anxious, they increase their avoidance of open arms on the EPM and central areas of a field arena (Hlinák et al. 2009; Mechiel Korte and De Boer 2003). The sucrose preference test (SPT), relies on the preference of animals for sucrose over water when given the option to drink either freely. An attenuation of this preference is usually interpreted as anhedonia or a loss of interest in previously enjoyed aspects of life during depressive episodes. Finally, for the purposes of this article, the forced swim task (FST) exploits the innate reaction of an animal to escape when placed in a vessel of water (Yankelevitch-Yahav et al. 2015). Eventually, it is inferred that the rat shows helplessness (Porsolt et al. 1977) by no longer attempting to escape from the vessel — hypothetically a passive coping behaviour (Yankelevitch-Yahav et al. 2015). In stressed rats, the latency to the onset of passive coping decreases and the time spent immobile versus swimming increases (Bogdanova et al. 2013). Such behaviour is typically compared with symptoms of despair and fatigue during depressive episodes in humans (American Psychiatric Association 2013; Carvalho et al. 2021).

A widely cited issue with MS is the enormous variability in the separation protocol used by different research groups (Schmidt et al. 2011; Tractenberg et al. 2016). Such variation may underlie the variability in behavioural outcomes caused by early separation stress in both rats (Schmidt et al. 2011) and mice (Tractenberg et al. 2016). A previous meta-analysis of the effect of MS in rats on anxiety measures (Wang et al. 2020) listed sex, length of separation, temperature during isolation, single versus whole litter isolation, treatment of the control group, and age of testing as the main sources of variation between different studies. In humans, an increased burden of childhood stress correlates with more severe mental health outcomes (Hughes et al. 2017). The parallel for this burden may be an extended period of separation of rat offspring throughout the preadolescent period. Earlier onset post-natal stress (i.e. separation starting at an earlier postnatal day) may also have a differential impact on behavioural outcomes (Leichtweis et al. 2020). We thus assessed not only the duration of separation but also the starting age of this procedure in our analysis of the MS protocol, in addition to examining sex-dependent differences. We also investigated the effects of ambient temperature on the behavioural outcomes of MS, which shows considerable variability across different studies (Harshaw and Alberts 2012; Melo et al. 2018; Zimmerberg and Shartrand 1992).

The impact of MS ultimately depends on how this stressor interacts with the hypothalamic–pituitary–adrenal (HPA) axis in developing pups (Smith et al. 1997; Stanton et al. 1988). These changes result in differential stress reactivity in rats as they age, implying that changes in behaviour seen after MS may be exacerbated following a second stress in adulthood (van Bodegom et al. 2017). Separated pups also show long-lasting changes in the response of the immune system to later stress in life (Dutcher et al. 2020). We therefore included studies that identified behavioural differences before and after a second stress in both MS and control groups. Our primary objective here was to identify common effects in unconditioned tasks for depression and anxiety phenotypes in rodents following MS. Our analysis also considered procedural differences in the MS protocol to establish the robustness of reported behavioural outcomes of animals exposed to ELS.

## Methods

### Search strategy

Relevant studies were identified by searching the online databases of PubMed and Web of Science on February 22nd, 2022. The following search criteria were used: (maternal separation) AND (Rats OR Rat) AND (Elevated Plus Maze OR Open Field Test OR Sucrose Preference Test OR Forced Swim Test). A manual search was also adopted to ensure all relevant studies were included in the meta-analysis. The search was not limited by year of publication.

### Study selection

Studies were included in the analysis if they conformed to the following criteria: (1) rats were used; (2) pups had been separated from their dams for at least 1 h daily for at least 7 days in the pre-weaning period. This differs from criteria in previous works that excluded studies that left their pups in the home cage during the separation period (Wang et al. 2020); (3) studies that measured depressive or anxiety-related behaviours on the EPM, OFT, SPT or FST; (4) studies that specified the sex of the animals, but unlike other approaches (Wang et al. 2020), if the data were pooled for both sexes the study was still included but separate analyses for pooled data were conducted; (5) studies were peer-reviewed and published in English; (6) studies reporting means ± SEM to allow the calculation of effect size.

**Fig. 1:**
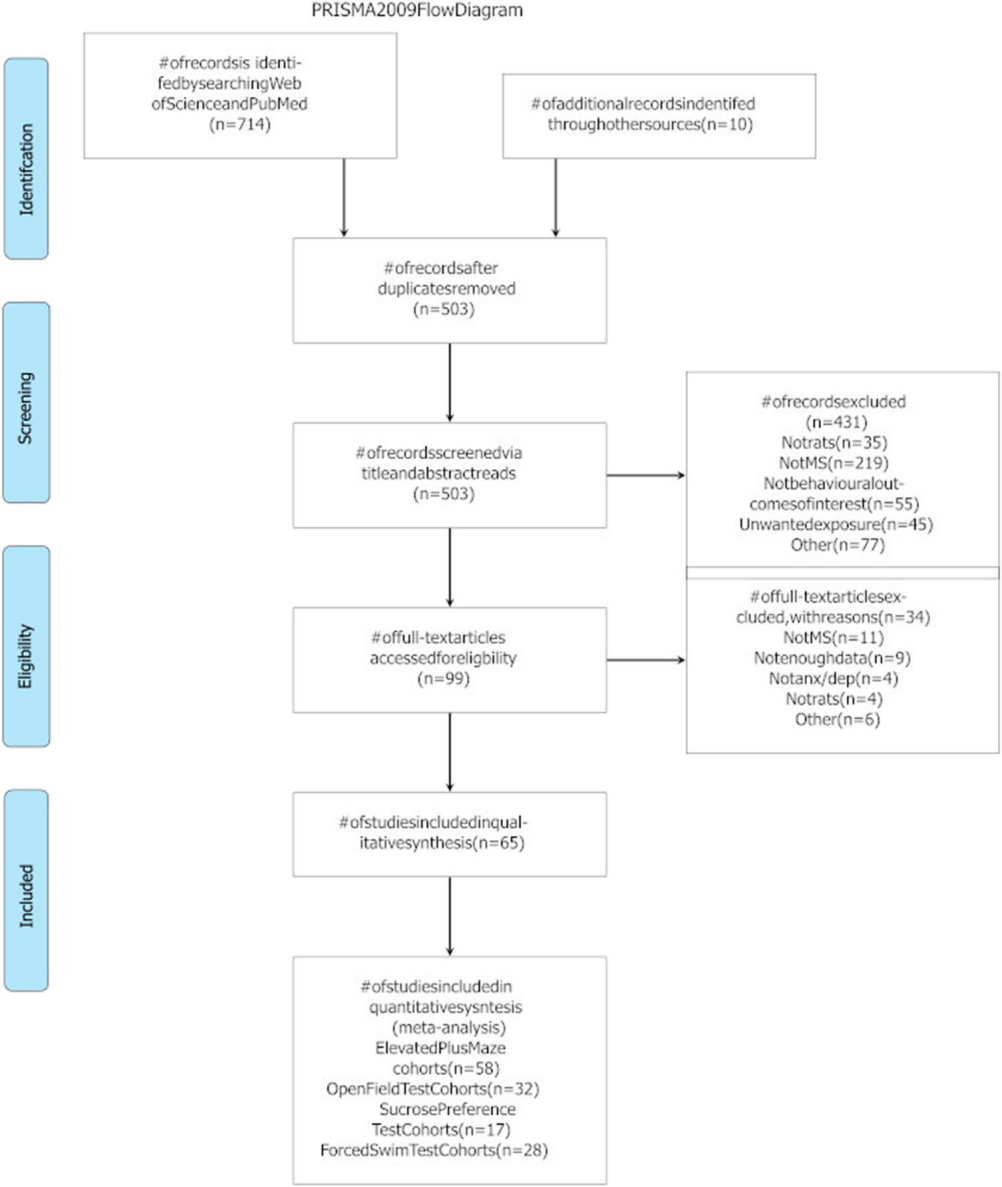
PRISMA flow diagram depicting the selection of studies

Figure 1 shows a PRISMA flow diagram indicating the study selection strategy. Over 700 studies were identified from searches with 65 studies deemed eligible for inclusion in the present meta-analysis. The 65 studies yielded 100 cohorts with at least 60 min separation from the dam on multiple days in early life. Thirteen cohorts that had been separated for 15 min only were identified but were not included in the present analysis. Across 36 papers, there were 58 cohorts of rats studied using the elevated plus maze (EPM) (*n* = 1364). Nineteen papers contributed a total 32 cohorts of rats tested in the open field test (OFT) (*n* = 793). Across 10 papers, there were 17 cohorts of rats studied using the sucrose preference test (SPT) (*n* = 416). Across twenty papers, 28 cohorts on the forced swim test (FST) (*n* = 561). Fifteen cohorts were tested on one or more of the behavioural outcomes of interest following a further stress later in life. A summary of the included studies and pertinent procedural characteristics is provided in Supplementary Material, Table 1.

### Data extraction

For each article, the following information was extracted: author; year of publication; rat strain; sex; presence of artificial heat source during separation; duration of each separation event in min; length of separation protocol in days; day of separation commencement; housing and treatment conditions of the control group; whole litter or individual pup separation; age at behavioural testing; whether a secondary adult stressor was applied, and the behavioural outcomes of each task (i.e. % of time in the open arm of the EPM, % time in the centre of the open field, % sucrose preference, time (s) immobile in the FST.

Behavioural outcomes were recorded as mean ± SEM for each subgroup. Where this was not explicitly available in the publication, data were extracted from graphs using the WebPlotDigitizer distance measurement function (Rohatgi 2021). Where one article contained multiple cohorts with different MS protocols, if available, the data were extracted and stored as a separate cohort. Where these different test cohorts had a common control group, the control group was split proportionally to avoid undue between-cohort correlations or the “unit-of-analysis” problem (Harrer et al. 2021: Chapter 3.5.2).

### Statistical analyses

Data analyses were carried out using R (R Core Team 2021) and meta (Balduzzi et al. 2019), metafor (Viechtbauer 2010) and dmetar ((Harrer et al. 2021): Appendix D). Hedge’s g effect size was calculated for the behavioural measures of interest, chosen for its robustness to small sample sizes in comparison to Cohen’s d. Confidence intervals were calculated using *t*-table values for studies where total *n* < 30 or z-values of ± 1.96 for larger samples. Pooled effect size (Hedge’s g) was calculated for each behavioural outcome using a random effects model. Knapp-Hartung adjustments (Knapp and Hartung 2003) were used to calculate the confidence interval around the pooled effect size. Heterogeneity was assessed using the Q test (Cochran 1954) and I ^2^ statistics (Higgins and Thompson 2002), though in general tests for heterogeneity are weak, especially with low numbers of studies in a typical meta-analysis (Ioannidis 2007). Random effects modelling was used over fixed effects even where estimated heterogeneity was low based on our hypotheses of variability. Publication bias was assessed using funnel plots and Egger’s test (Egger et al. 1997). Moderators of interest were either categorical or continuous. Categorical moderators were assessed using χ^2^ test for subgroup differences. Continuous moderators, following collinearity analysis, were fitted by forced entry to a meta-regression using a mixed effects model using maximum likelihood for τ estimator. Then significant moderators, as analysed by subgroup analysis, were entered into the model and moderators removed by backwards stepwise regression, removing all moderators where *p* > 0.05 until only significant moderators remained. It is noted that stepwise models increase the likelihood off overfitting (Chatfield 1995; Whittingham et al. 2006) but our low number of moderators and precedent for use of this model (Wang et al. 2020) balances this concern. Studies that included exposure to an adult stress were treated as a subgroup; as such χ^2^ test for subgroup differences was applied.

## Results

### Variability in MS protocols

Of the included studies, the most common experimental design was the separation of pups for 3 h, each day, starting from PND 2 to PND 4. When separated from their dams, pups were kept with the rest of their litter in a temperature-controlled environment, in a different experimental room to the dam. The separated group was most often compared with a control group that had standard animal facility rearing — though this differed from study to study and was marginally more common than a control group that was entirely undisturbed. There was no significant collinearity between the three continuous predictors, thus all were used in each primary meta-regression. There were 7 identified secondary stressors, the most used was a variable chronic stress paradigm. All secondary stressors were applied following weaning, most often around PND 50.

### Small or non-existent effects of MS on unconditioned behavioural measures

**Fig. 2:**
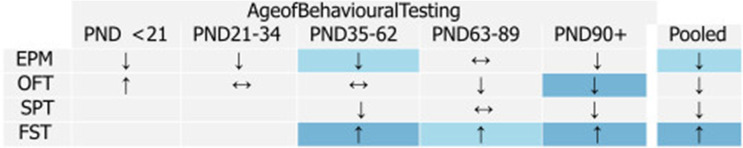
Effect size trends for different age group on each behavioural task. A down arrow indicates a decrease in performance of that measure in MS animals compared with controls. Uncoloured cells represent effect sizes that have confidence intervals including zero. Light blue cells indicate effect sizes that have confidence intervals that do not contain zero. Dark blue cells indicate effect sizes that have confidence intervals that do not contain zero and have a large effect size (greater than 1 or less than − 1). Left/right arrows indicate effect sizes within 0.2 of zero

**Fig. 3:**
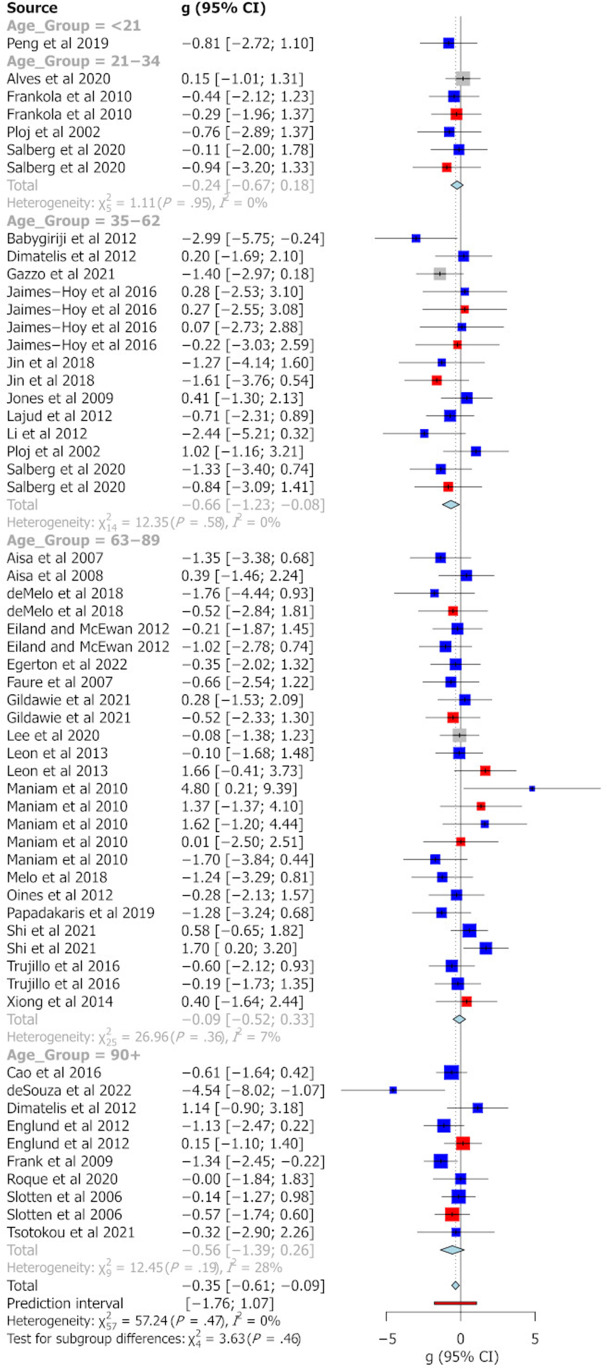
Forest plot showing a meta-analysis of the effect of MS on % of time in open arms of the EPM. Blue boxes indicate studies in males, red are studies in females and grey boxes indicate studies that had pooled sex analysis. The Hedge’s g is depicted for each individual cohort with 95% CI, labelled on the left. The pooled Hedge’s g calculated by random effects model is displayed for each age group with a diamond and total for all cohorts at the bottom of the plot. The red bar indicates the overall prediction interval. The size of the box for individual cohorts correlates with the weighting attributed by the REM for the pooled g

**Fig. 4:**
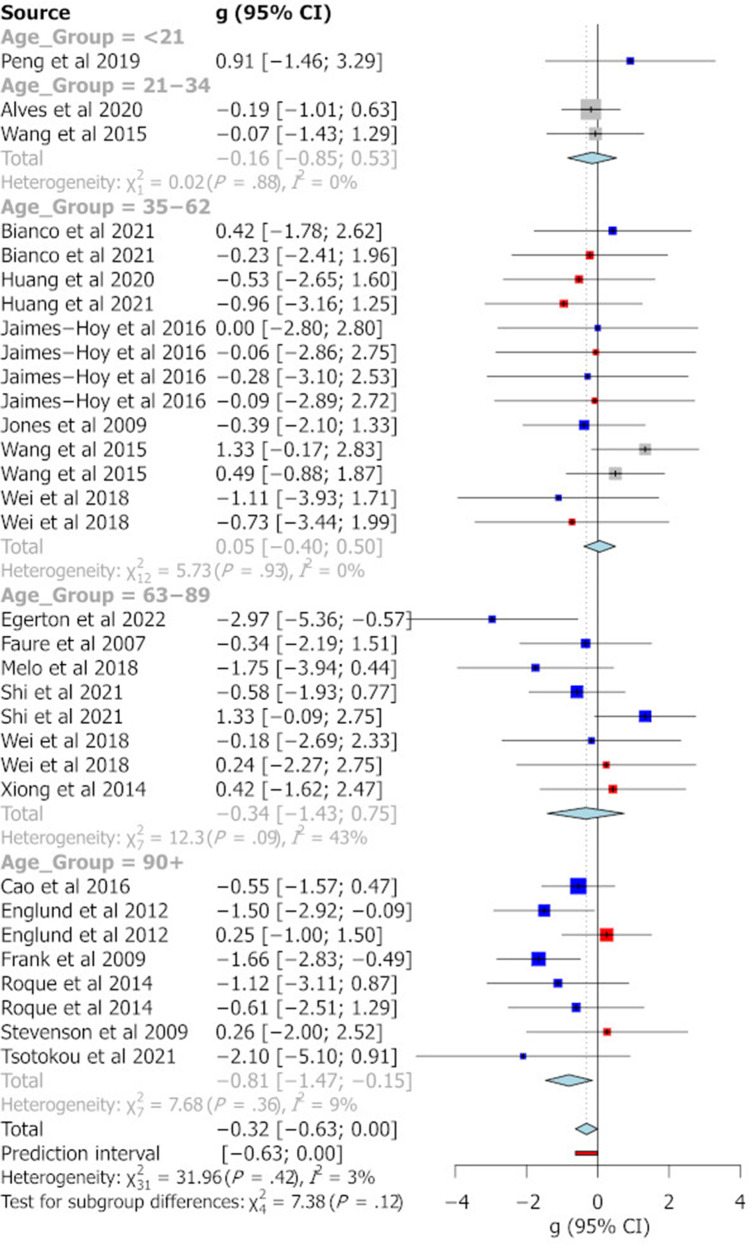
Forest plot showing a meta-analysis of the effect of MS on % of time in open areas of the OFT. Blue boxes indicate studies in males, red are studies in females and grey boxes indicate studies that had pooled sex analysis. The Hedge’s g is depicted for each individual cohort with 95% CI, labelled on the left. The pooled Hedge’s g calculated by random effects model is displayed for each age group with a diamond and total for all cohorts at the bottom of the plot. The red bar indicates the overall prediction interval. The size of the box for individual cohorts correlates with the weighting attributed by the REM for the pooled g

**Fig. 5:**
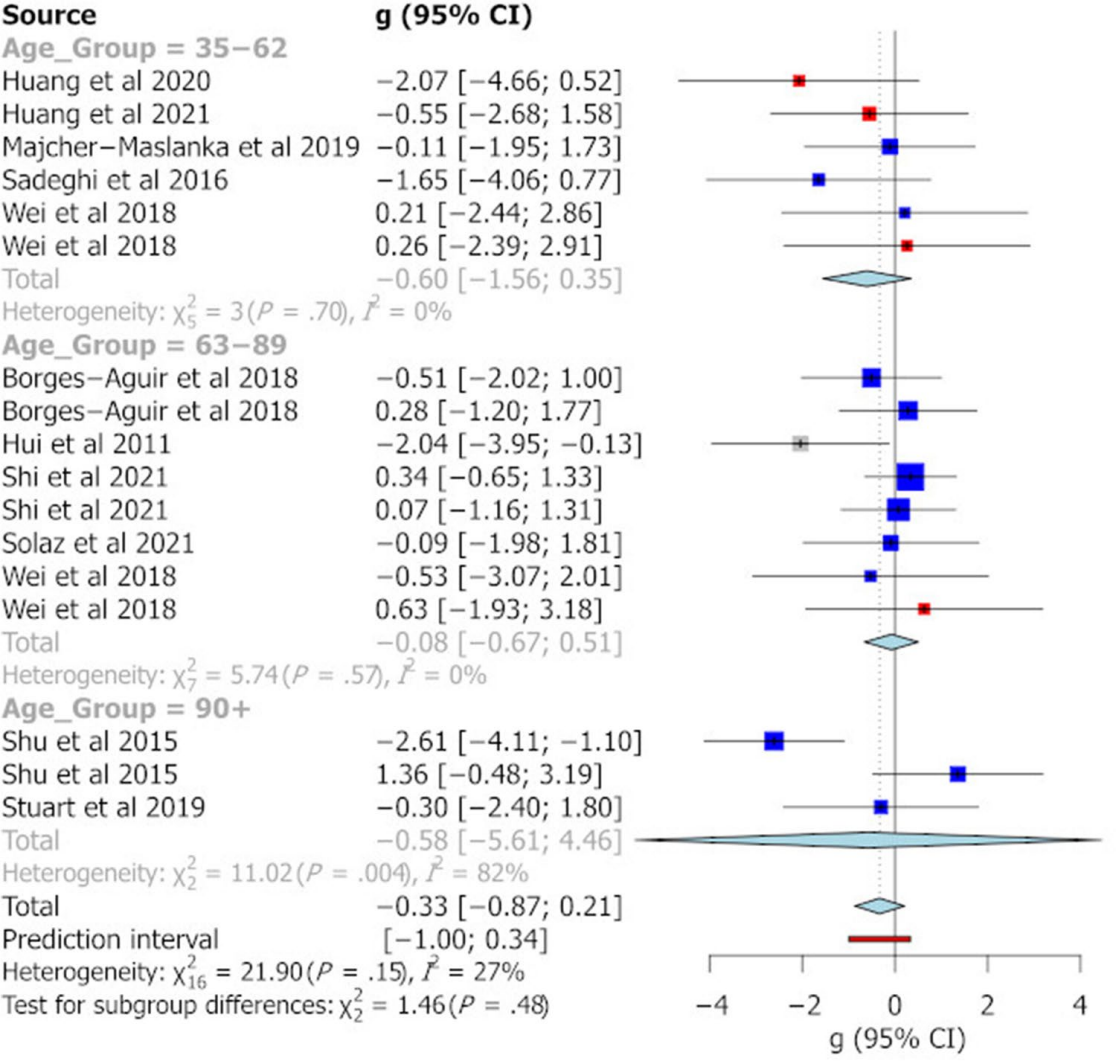
Forest plot showing a meta-analysis of the effect of MS on % sucrose preference. Blue boxes indicate studies in males, red are studies in females and grey boxes indicate studies that had pooled sex analysis. The Hedge’s g is depicted for each individual cohort with 95% CI, labelled on the left. The pooled Hedge’s g calculated by random effects model is displayed for each age group with a diamond and total for all cohorts at the bottom of the plot. The red bar indicates the overall prediction interval. The size of the box for individual cohorts correlates with the weighting attributed by the REM for the pooled g

**Fig. 6:**
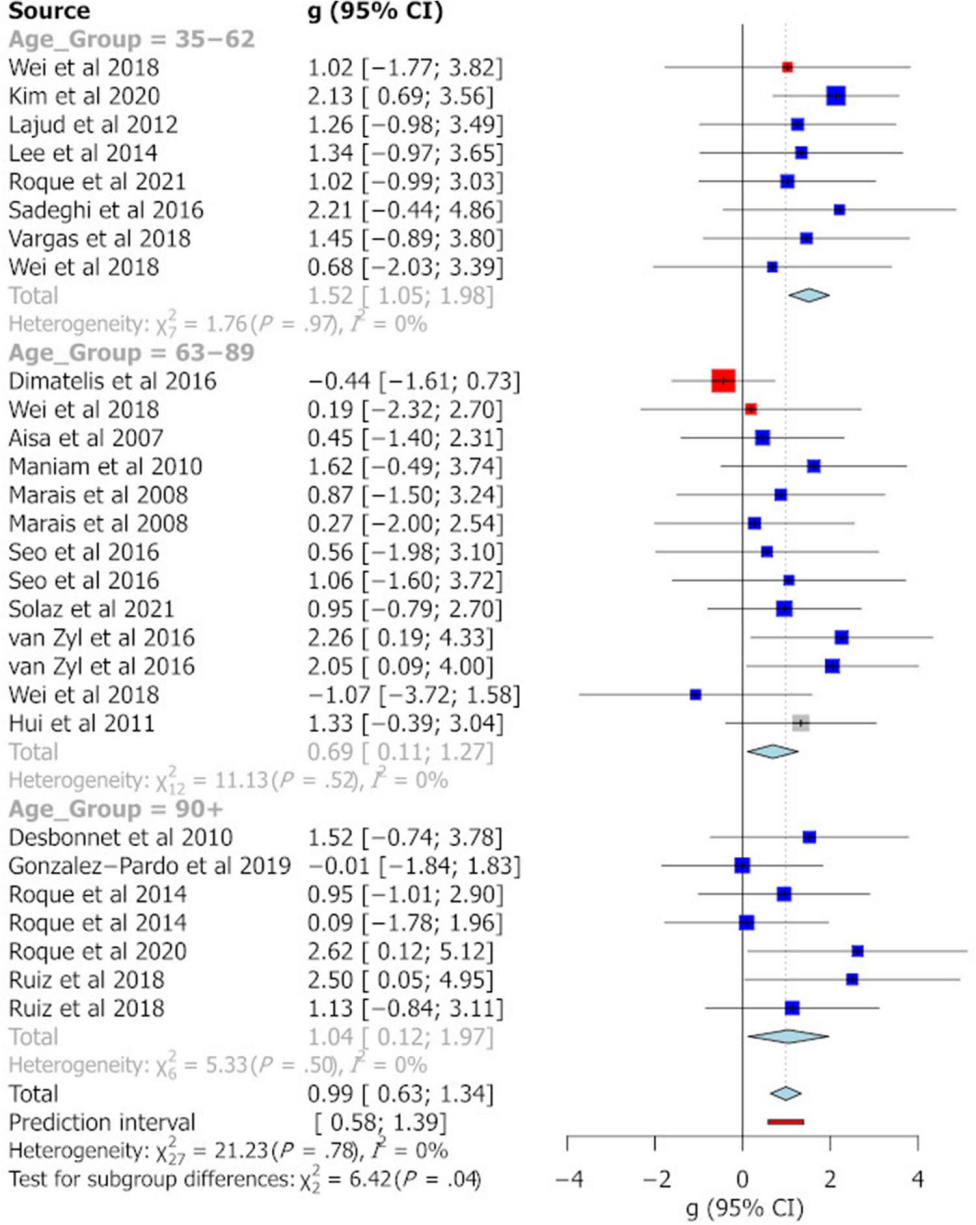
Forest plot showing a meta-analysis of the effect of MS on immobility time in the FST. Blue boxes indicate studies in males, red are studies in females and grey boxes indicate studies that had pooled sex analysis. The Hedge’s g is depicted for each individual cohort with 95% CI, labelled on the left. The pooled Hedge’s g calculated by random effects model is displayed for each age group with a diamond and total for all cohorts at the bottom of the plot. The red bar indicates the overall prediction interval. The size of the box for individual cohorts correlates with the weighting attributed by the REM for the pooled g

The categorisation of age groups was defined on the basis of previous work indicating major developmental age ranges in the rat (McCutcheon and Marinelli 2009). Figure 2 summarises the effect sizes reported for different age groups across the different behavioural tasks analysed in this study. Pooled Hedge’s g statistics indicate significant behavioural differences between MS and control animals for the EPM (Fig. 3) and FST (Fig. 6) only [EPM g =  − 0.35 (95% CI =  − 0.61, − 0.09), *t* =  − 2.69, *p* = 0.0095, k = 58; FST g = 0.99 (95% CI = 0.63, 1.34), *t* = 5.64, *p* = 0.0001, k = 28]. Hedge’s g values for individual cohorts varied widely for each task. In the EPM, individual Hedge’s g clustered around 0 and had the largest prediction interval (see Fig. 3, prediction interval: − 1.76, 1.07). All effect sizes calculated were small (0.2 < g < 0.5) or moderate (0.5 < g < 0.8) except for the pooled g for FST where in peri-adolescents and older adults and OFT in older adult rats, a large effect size not including 0 was identified (see Fig. 4). In relation to the SPT, reported effect sizes across all age groups were not significant (see Fig. 5). In addition, there was no significant evidence of publication bias for any of the behavioural measures, as indicated by the symmetrical funnel plots and concordant non-significant Egger’s test values (see S2).

### Lack of effect of methodological variables on behavioural outcomes of MS

I ^2^ values for all the behavioural tasks were not significant, thereby indicating that variability in reported effect sizes was not due to true differences in subgroup effect sizes. This conclusion was supported by a moderator analysis where no significant effect of subgroup was noted for the reported effect sizes for any of the behavioural tasks, both in subgroup χ^2^ analysis and meta-regression. Nevertheless, for the OFT and FST, the difference between MS and controls was greater in males than in females [OFT: male g =  − 0.67 (95% CI =  − 1.16, − 0.18), female g =  − 0.05 (95% CI =  − 0.39, 0.27), χ2 = 6.59, *p* = 0.04][FST: male g = 1.17 (95% CI = 0.82, 1.54), female g =  − 0.15 (95% CI =  − 1.68, 1.37), χ2 = 11.58, *p* = 0.003]. The date of first maternal separation was the only continuous moderator that had a significant impact on effect size in meta-regression. An earlier start day for MS predicted a greater effect size on the EPM (β = 0.44, se = 0.16, (95%CI = 0.12, 0.76), *t* = 2.73, *p* = 0.008), an effect that was robust to permutation tests (p* = 0.007) but was not demonstrated for any other task (Fig. 6).

### Effect of a second uncontrollable stressor in MS animals

**Fig. 7:**
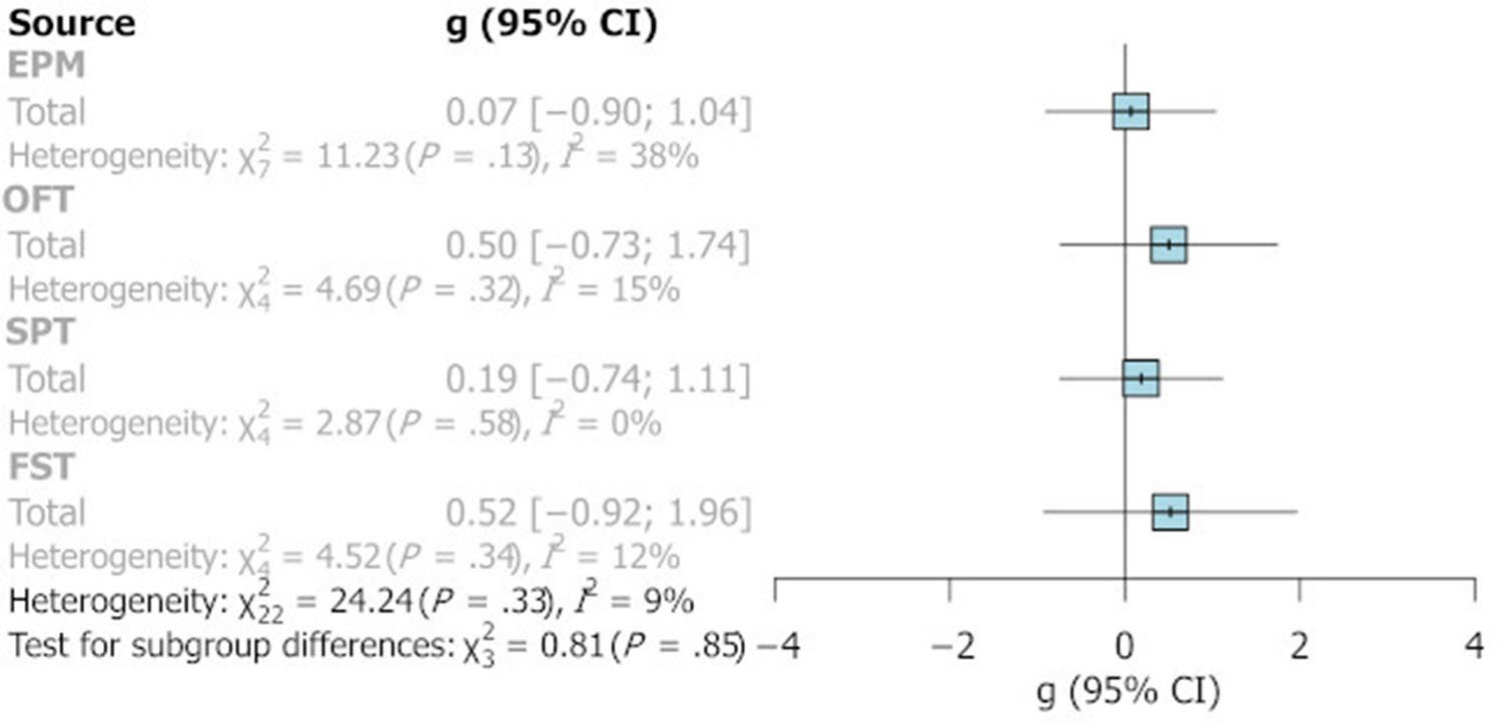
Summary forest plot illustrating pooled Hedge’s g for cohorts subjected to a secondary stress following MS. Labels on the left identify the task. The g is shown with a 95% CI

Seventeen cohorts were exposed to a second stress and these varied both in nature and timing. Cohorts were mostly exposed to multiple day variable chronic mild stress, or chronic restraint stress, though some cohorts used alternate stresses such as isolation and sleep deprivation. The day of application of second stress was predominantly in young adulthood and varied from PND 26 to PND 91. A detailed subgroup analysis failed to identify a significant difference in the reported effect sizes between cohorts tested with or without a secondary stress in adulthood. Thus, for the EPM, OFT and SPT effect sizes clustered around g = 0 with wide CIs (see Fig. 7). A larger effect size was observed for the FST but was also accompanied by a large CI that encompassed zero.

## Discussion

As far as we are aware, this is the first meta-analysis of spontaneous behaviours claimed to assess depression- and anxiety-like phenotypes in adult rats exposed to repeated early maternal separation. Sixty-five studies were selected, encompassing one hundred cohorts across four widely used behavioural paradigms. Repeated maternal separation is a widely used experimental procedure to investigate the consequences of early life stress with long lasting behavioural (Nishi 2020), immune (Dutcher et al. 2020) and neurobiological (Nishi 2020) effects. Given the enormous burden of depression and anxiety-related disorders in humans (Bromet et al. 2011; Global Health Data Exchange, n.d.; WHO 2022), it has never been more important to develop robust behavioural procedures in animals to investigate underlying neurobiological mechanisms to help inform the development of new treatments.

The main findings of our analysis indicate that in general unconditioned tasks are not robustly sensitive to behavioural phenotypic changes in rats exposed to MS. Nevertheless, significant effect sizes were detected for the EPM and FST (g =  − 0.35; g = 0.99 respectively), and therefore these two tasks should be included as part of a battery of tests to investigate elements of depressogenic and anxiogenic phenotypes associated with maternal separation stress. However, the FST in particular faces challenges of power and validity (Reardon 2019; RELACS Consortium et al. 2021) and we would not use the large effect size seen here to justify its continued use. Moreover, many of the moderators of interest did not explain the variability in effect sizes across the different cohorts. Although the literature regarding the impact of a second stress in MS animals is sparse, we found no evidence that the four tasks analysed in the present study are sufficiently sensitive to detect differential impacts of a secondary stressor in MS animals.

### Unconditioned measures are not robust behavioural validations of maternal separation

As noted above, sample sizes in preclinical studies tend to be small (Vesterinen et al. 2014), with limited power to detect differences with small effect sizes, such as found here in the EPM. This was particularly the case for studies that have used the EPM where our analysis unequivocally demonstrated that this procedure is unsuitable as a behavioural screen for animals according to their previous history of stress. The utility of the EPM for mechanistic investigations into the neurobehavioural sequelae of maternal separation is therefore limited to a single observational time-point of unconditioned anxiety-like behaviour. Emphasis should be placed on baseline preference for safer environments rather than nuanced interpretations of the anxiety around open environments; especially given the lack of correspondence of this measure with the OFT. The EPM is underpinned by an ethological fear of heights and open spaces and therefore the task itself is likely to be stressful for the animal. Therefore, when using the EPM within a battery of tasks the effect of a stressful experience on other tasks should be duly noted.

The FST is also widely used to characterise stress-related disorders, notably depression-like behaviour in rodents (Carvalho et al. 2021; Yankelevitch-Yahav et al. 2015). Our analysis demonstrates the utility of this task in identifying meaningful differences between controls and maternally separated animals. However, growing concerns about the construct validity of this procedure have discouraged funding bodies and the pharmaceutical industry from continuing to invest in research that adopts this task (Reardon 2019). The main argument for the utility of this task in fact usually rests with predictive validity. Thus, drugs with clinical efficacy in depression such as SSRIs increase swim time and delay the onset to passive coping (Yankelevitch-Yahav et al. 2015). However, this beneficial effect results from acute systemic administration and does not resemble the much slower onset of clinical efficacy in humans (Thompson 2002). Although the FST exhibits sensitivity in identifying anti-depressant like compounds, especially compounds that modulate the monoaminergic systems (Cryan et al. 2005), not all compounds that reduce immobility times have anti-depressant activity and consequently fail in clinical trials (Bowman and Daws 2019; Cryan et al. 2005; Cryan and Mombereau 2004). Moreover, a more mixed response is observed with alternative anti-depressant therapies (Yankelevitch-Yahav et al. 2015). As questions of validity grow, so do ethical concerns of performing such an aversive task in animals (Reardon 2019).

Previous summary analysis in rodents has only addressed anxiolytic tests, they found a significant impact on EPM and OFT in rats (Wang et al. 2020). The effect sizes reported are comparable to those identified for the same behavioural tasks in this current work, yet we did not identify most of these effect sizes to be significant. This suggests that the literature added due to wider inclusion criteria and date of publication in this current study has a similar overall effect size as identified in Wang et al. (2020) but increased variation in reported effect size reduces the significance of the results, calling into question if these effect sizes represent meaningful behavioural differences following MS.

Rather surprisingly, the SPT returned the smallest effect size and highest variability of the measures analysed in this study. The SPT is claimed to assess anhedonia, but sucrose preference per se does not reflect ingestive behaviour associated with depressive episodes in humans with depression (Criteria 3 depression symptoms: American Psychiatric Association 2013; Simmons et al. 2020). Moreover, DSM-V criteria for ‘disinterest in previously liked activities’ is not limited to food preferences (American Psychiatric Association 2013). Nevertheless, the SPT has still been considered the gold standard procedure for the assessment of anhedonia (Bacharach and Calu 2019; Tõnissaar et al. 2006) and is routinely used in pre-clinical research (Cui et al. 2020a; Dallé et al. 2020; Jiang et al. 2021), despite its dependence on subjective ‘face validity’. Although our findings question the utility of this task in the behavioural assessment of ELS, we acknowledge that conditioned tests of affective disorder are often motivated by food reward (Bari et al. 2010; Enkel et al. 2010; Harding et al. 2004; Papciak et al. 2013; Phillips et al. 2018). Indeed, MS rats tested during adulthood show an attenuation of food conditioned anticipatory locomotor activity (Matthews et al. 1996). Taken together, MS appears to cause long lasting changes in incentive learning processes rather than the consumption of, or preference for, palatable food.

### Lack of effect of maternal separation on unconditioned behaviours is not due to methodological variability

As there is no standard protocol for MS, it is not surprising that considerable variability exists in the precise way this procedure is carried out. Overall, most of the moderators of interest, based on prior findings, did not account for the variability in the calculated effect sizes. Thus, the variables of sex, age and MS start day were the only moderators across the four tasks to affect behavioural outcomes, but their impact was not consistent across tasks. Our analysis did identify that MS start day has a significant effect on the predicted Hedge’s g for EPM following MS, indicating that the time during development that a stressor is applied may create a more anxiogenic phenotype. Our analysis identified that the start date of MS has a significant effect on the predicted Hedge’s g for the EPM task following MS. However, this observation is tempered by the analysis of Wang (2020) where separation duration, rather than start date, was a significant moderator of MS outcomes. Although collinearity analysis did not indicate significant moderator co-dependence, it is reasonable to assume that both moderators represent the intensity of MS stress. We interpret these findings to suggest that EPM may be useful at identifying phenotypic differences following maternal separation stress but the sensitivity of this procedure may depend on the severity of the stress, including the start time (PND < 2) and duration of separation.

We calculated the I^2^ heterogeneity statistic or the proportion of the variability in observed effect sizes that was due to variability in the true effect size rather than random error (Borenstein et al. 2017). Since our reported effect sizes did not vary significantly, a low I^2^ value implies that any variability was due to random error rather than the precise parameters of the MS procedure.

### Beyond unconditioned measures of depression and anxiety

In this section, we discuss alternative behavioural tasks to assess depression and anxiety-related phenotypes in rats previously exposed to ELS. These tasks are generally computer automated as well as objectively scored and based on analogous tasks in humans that assess comparable psychological processes, thereby facilitating translational research. Though these tasks have been used less often than traditional unconditioned tests, and therefore we cannot yet conduct a meta-analysis of this scale to assess their utility, Table 1 provides a summary of the main tasks in common use that fall within this category for discussion of their potential. A significant limitation of conditioned behavioural tasks is that they often require weeks of pre-training before the test phase and thus where unconditioned tasks may provide information about the acute phenotypic state, conditioned tasks are more useful to assess parameters such as learning rate, change in performance over time and acute changes in performance following life events.

**Table 1:** Contemporary behavioural tasks to assess depression-related phenotypes in rodents

Task	Procedural details	Relevance to depression	References
Ambiguous Cue	Training to distinguish between two tones with distinctly different outcomes. Test phase is a combination of training tones and ambiguous tones. Key outcomes are the proportion of optimistic responses to the ambiguous tone (responses directed to the more highly rewarded tone) and response latencies	Negative bias or lack of positive bias. Indicated by greater response omissions or pessimistic responses to the ambiguous cues. May also be associated with increased response latencies	Enkel et al., 2010 Harding et al., 2004 Papciak et al., 2013 Stuart et al., 2019
Probabilistic Reversal Learning	Training to distinguish between two cues with different reward contingencies. One is more often rewarded – e.g. an 80:20 probabilistic reinforcement protocol. The contingency reverses after some trials in the test phase. Typical dependent variables include response latencies, win-stay probability, lose-shift probability, number of reversals, perseverative responses	Win-stay proportion decreases, increased response latencies reflecting impaired decision-making. Blunted reward-related learning	Bari et al., 2010 Philips et al., 2018

### Ambiguous cue task

Ambiguous cue tasks are increasingly used in humans and experimental animals (Enkel et al. 2010; Gethin et al. 2017; Harding et al. 2004; Papciak et al. 2013; Stuart et al. 2019; Surguladze et al. 2004) to assess negative affective bias and slowed processing speed in major depressive disorder (MDD), especially where individuals are exposed to ELS (Saleh et al. 2017). In humans, depressed and control individuals identify positive, negative, and neutral emotional faces with equivalent accuracies and response latencies (Gur et al. 1992; Hale 1998; Surguladze et al. 2004) but when presented with ambiguous face cues depressed individuals are more likely to label positive ambiguous faces as neutral and near-negative faces as negative (Surguladze et al. 2004). This shift in positive bias is pervasive through immediate processing to recall of episodic memory (Gethin et al. 2017). Furthermore, MDD patient populations with a history of ELS are more likely to lack a positive affective bias compared to both MDD without ELS and controls (Gethin et al. 2017) during and between depressive episodes. In humans, a lack of positive affective bias is thought to represent a risk marker for depression (Gotlib and McCann 1984; Hales et al. 2014).

Analogous ambiguous cue tasks have been developed in experiment animals. Positive and negative valence can be assigned to individual auditory cues that are linked to an appetitive task representing gain of reward or avoidance of punishment during training (Enkel et al. 2010; Harding et al. 2004; Papciak et al. 2013; Stuart et al. 2019). Rodents with a history of stress are slower to respond to ambiguous auditory cues (Harding et al. 2004) and show a negative affective bias (Enkel et al. 2010; Harding et al. 2004; Papciak et al. 2013). When investigating the effects of stress in adulthood on depression, the use of punishment in this task could result in the task itself acting as a secondary physiological stressor. In this situation, an altered judgement bias task may be more useful whereby the valence of the options are both positive, but one is more positive (Hales et al. 2016, 2021). This ‘pessimistic’ expectation of rodents when faced with an ambiguous cue is seen following a variety of models to induce stress phenotypes (Enkel et al. 2010; Harding et al. 2004; Papciak et al. 2013; Stuart et al. 2019). Notably, the MS procedure is sufficient to induce this pessimistic phenotype on an ambiguous cue task (Stuart et al. 2019). MS rats are less able to integrate rewarding information to adjust biases (Stuart et al. 2019) indicating a lack of positive bias as opposed to a negative affective bias. This more closely mirrors the findings in MDD and humans with an experience of ELS. Further support for the translatability of this task is limited by the investigation of pharmacological intervention focussing on acute dosing. Acute systemic administration of SSRIs such as citalopram and escitalopram produce dose-dependent shifts in bias as assessed using ambiguous cue tasks (Drozd et al. 2019; Rygula et al. 2014), with the tricyclic monoamine inhibitor desipramine also inducing a shift in negative bias, but d-amphetamine at higher doses producing a positive bias (Rygula et al. 2014). With respect to the PRL task, selective brain 5-HT depletion (Bari et al. 2010) and 5HT2C receptor antagonism (Phillips et al. 2018) leads to a decrease in the proportion of win-stay responses. Moreover, the atypical antidepressant agomelatine decreased loss-shift behaviour in the PRL and ambiguous cue tasks, suggesting that treated animals were more robust to negative feedback (Drozd et al. 2019). Such effects in the ambiguous cue task were only apparent in animals identified as trait “pessimistic” (Drozd et al. 2019) suggesting that drug effects may be importantly modulated by baseline differences in lose-shift / win-stay behaviour.

### Probabilistic reversal learning

Integration of reward-based information is an integral component of probabilistic reversal learning (PRL) tasks. PRL measures cognitive flexibility in the context of decision-making and the ability to integrate fluctuations in reward likelihood in both humans and animals (Bari et al. 2010; Mukherjee et al. 2020; Murphy et al. 2009; Phillips et al. 2018; Taylor Tavares et al. 2008). PRL tasks are used in a clinical setting to assess the cognitive symptomology in depression states (Murphy et al. 2009; Taylor Tavares et al. 2008). Patients with MDD are slower to learn and less responsive to reversals on PRL tasks (Mukherjee et al. 2020; Wilkinson et al. 2021). In addition, individuals with a history of ELS show reduced responsiveness to positive feedback and reduced positive reinforcement learning for high magnitude rewards (Wilkinson et al. 2021).

Rodents can be trained on PRL tasks that are remarkably similar to those used in human participants. Two cues can be presented to the rats and correct responses corresponding with selecting the cue with the higher probability of reward. Following a criterion having been attained of correct responses, stimulus-reward contingencies are reversed, which allows for a number of behavioural measures to be assessed including the latency to respond following a contingency reversal and the proportion of correct win-stay or loss-shift responses when the outcome does not match the expected return. Computational modelling based on reinforcement learning theory can also be applied to both human and experimental animal performance on the PRL task (Robbins and Cardinal 2019). Pharmacological models of depression in rats have elucidated permutations in PRL behaviour in rats (Bari et al. 2010; Phillips et al. 2018). The proportion of win-stay responses is decreased following serotonin depletion (Bari et al. 2010) and 5HT2C receptor antagonism (Phillips et al. 2018) highlighting in rats the same decreased attention to positive reinforcement identified in humans (Wilkinson et al. 2021). Interesting work using both the PRL and the ambiguous cue task indicates that administration of the atypical antidepressant agomelatine reduces loss-shift behaviour, indicating they are more robust to negative feedback, but only in animals identified as trait “pessimistic” using the ambiguous cue (Drozd et al. 2019). This work supports our general suggestion that combinations of tasks can be used to describe both cognitive changes in depressogenic models and to explain individual differences in susceptibility to certain types of pharmacological intervention. Unpublished data using the PRL task in rodents who have been exposed to MS indicates ELS has a long-lasting impact on the response latency in PRL (Dutcher E, unpublished findings) and this is exacerbated by later life stress. MS is sufficient to induce PRL behavioural changes, but a “double-hit” with a later life stress exacerbates these effects. There are also sexually dimorphic sensitisations to negative outcomes to influence learning which indicate the potential of the PRL task in ELS rats to illustrate the reinforcement learning blunting seen in humans (Wilkinson et al. 2021).

### Concluding remarks

The present meta-analysis has drawn on a large body of empirical evidence using raw means to calculate effect sizes across many cohorts. While most studies analysed contained all the procedural details we wished to extract, maximizing power for moderator analysis, including of a wide literature base in the pooled analysis allowed for robust conclusions to be drawn on the utility of measuring spontaneous behaviours for the assessment of anxiety and depression phenotypes. Where Wang (2020) identified evidence of potential publication bias (i.e. the tendency to not publish negative results) particularly for studies using EPM, we did not. This could explain why they previously noted a significant and moderate effect size for cohorts tested on EPM, and we did not, if our inclusion criteria increased the proportion of studies with null and negative findings. Further, our lack of evidence of publication bias indicates appropriate use of inclusion criteria for this meta-analysis such that studies that more likely had null and negative results were not disproportionately excluded. We included cohorts of both sexes and had a large sample size for both males and females. Nevertheless, a few limitations in our analysis should be noted. For example, a lack of observable pooled effect size across the tasks limited our ability to analyse moderator effects and the impact of a second stressor in adulthood after MS. In particular, the small number of cohorts that applied a second stressor in adulthood further limited this assessment. A further limitation of this analysis is that whilst wide inclusion criteria allow for a broad overview of the literature, we did not include qualitative assessments of the included studies. We also note that while we have suggested some alternate behavioural metrics to assess the depression phenotype, there are still too few studies using contemporary methods such as automated touchscreen tasks to make strong claims regarding their usefulness. Conditioned tasks such as the ambiguous cue and PRL are not being proposed here as like-for-like replacements of unconditioned tasks but have the advantage of revealing cognitive processes less obviously identifiable with unconditioned tasks.

Research on mental health is increasingly focused on mechanisms to guide treatment options, overturning the historical search for new drugs on the back of old drugs. To facilitate the pursuit for relevant biomarkers, including those relating to stress reactivity, robust experimental approaches and behavioural tasks are needed in experimental animals. Although this study was unable to verify any task as particularly suitable for behavioural screening, our analysis highlights variation in MS protocol as a moderator of effect size. In line with contemporary inclusion criteria, both males and females should be included in MS studies, especially as *sex* was the only moderator in our hands to impact effect size in more than one task.

In conclusion, the present meta-analysis of four unconditioned tasks indicates that the effect sizes for depression- and anxiety-like phenotypic outcomes of ELS are small and highly variable. Although the FST reveals significant difference between control and separated animals, the underlying neurobiology that mediates this effect is poorly understood. Given the additional poor translatability of unconditioned tasks to MDD in humans, we suggest that any further use of unconditioned tasks is within a battery of tasks that assess conditioned and unconditioned behaviours and are not relied upon in isolation. The MS procedure however remains a valuable experimental approach for revealing the underlying neurobehavioural mechanisms of ELS (Schmidt et al. 2011; Tractenberg et al. 2016). Looking forward, translational preclinical research incorporating automated operant or instrumental procedures, including touchscreen tasks measuring different aspects of goal-directed behaviour, promise exciting new ways of exploring persistent behavioural sequelae of MS and impacts of future uncontrollable stressors.

### Supplementary Information

Below is the link to the electronic supplementary material.

Supplementary file1 (PDF 44 KB)

Supplementary file2 (DOCX 181 KB)
