# Supplementary material for: “The wrong tools for the right job”: a critical meta-analysis of traditional tests to assess behavioural impacts of maternal separation
Source: Psychopharmacology (Berl). 2022 Nov 23;240(11):2239–56. doi: 10.1007/s00213-022-06275-6 (PMC10593619; doi:10.1007/s00213-022-06275-6)
Supplement: Supplementary file 1 — Supplementary file1 (PDF 44 KB) [file 213_2022_6275_MOESM1_ESM.pdf]

Author	Sex	Format of Separation	Control	Age of First Test	Second Stress	Behavioural Test
Aisa et al., 2007	M	P2-P21, 3h/day, T, W	AFR	P60		EPM, FST
Aisa et al., 2008	M	P2-P21, 3h/day, T, W	AFR	P60		EPM
Alves et al., 2020	MF	P2-P14, 3h/day, T, W	AFR	P33		EPM, OFT
Babygirija et al., 2012	M	P2-P14, 3h/day, T, W	Undisturbed	P56		EPM
Bianco et al., 2021 M	M	P2-P14, 3h/day, T	AFR	P38		OFT
Bianco et al., 2021 F	F	P2-P14, 3h/day, T	AFR	P38		OFT
Borges-Aguiar et al., 2018	M	P2-P21, 3h/day, T, W	Handling	P59		EPM, SPT
Borges-Aguiar et al., 2018	M	P2-P21, 3h/day, T, W	Handling	P59		EPM, SPT
Cao et al., 2016	M	P2-P21, 3h/day, I	Undisturbed	P90		EPM, OFT
Cui et al., 2020 M	M	P1-P21, 3h 2X/day, I, W	Undisturbed	P56		
Cui et al., 2020 F	F	P1-P21, 3h 2X/day, I, W	Undisturbed	P56		
de Melo et al., 2018 M	M	P3-P14, 3h/day, T, W	Undisturbed	P87		EPM
de Melo et al., 2018 F	F	P3-P14, 3h/day, T, W	Undisturbed	P87		EPM
de Souza et al., 2022	M	P2-P14, 6h/day, ,W	Half	P120		EPM
Desbonnet et al., 2010	M	P2-P14, 3h/day, T, W	AFR	P90		FST
Dimatelis et al., 2012	M	P2-P14, 3h/day, T, W	AFR	P40		EPM
Dimatelis et al., 2012	M	P2-P14, 3h/day, T, W	AFR	P99	Prior Testing	EPM
Dimatelis et al., 2016	F	P2-P14, 3h/day, T, W	AFR	P67		FST
Eiland and McEwen, 2012	M	P2-P12, 3h/day, T, W	AFR	P80		EPM
Eiland and McEwen, 2012 s	M	P2-P12, 3h/day, T, W	AFR	P80	Single Restraint	EPM
Egerton et al., 2022	M	P2-P12, 3h/day, T, W	Undisturbed	P70		EPM, OFT
Englund et al., 2021 M	M	P2-P14, 3h/day, T, W	Undisturbed	P90		EPM, OFT
Englund et al., 2021 F	F	P2-P14, 3h/day, T, W	Undisturbed	P90		EPM, OFT
Faure et al., 2007	M	P2-P14, 3h/day, T, RT	AFR	P67	Triple Stressor	EPM, OFT
Frank et al., 2019	M	P1-P20, 6h/day	Undisturbed	P90		EPM, OFT
Frankola et al., 2010 M	M	P2-P14, 3h/day, T, W	MS15	P26		EPM
Frankola et al., 2010 F	F	P2-P14, 3h/day, T, W	MS15	P26		EPM
Gazzo et al., 2021	MF	P2-P12, 3h/day, T, W	Undisturbed	P45		EPM
Gildawie et al., 2021 M	M	P2-P20, 4h/day, I, W	AFR	P70		EPM
Gildawie et al., 2021 F	F	P2-P20, 4h/day, I, W	AFR	P70		EPM
González-Pardo et al., 2019	M	P1-P22, 4h/day, T, W	AFR	P90		FST
Huang et al., 2021	F	P1-P21, 3h 2X/day, T, RT	Undisturbed	P56		OFT, SPT
Huang et al., 2021 s	F	P1-P21, 3h 2X/day, T, RT	Undisturbed	P56	VCS	OFT, SPT
Hui et al., 2011	MF	P2-P14, 3h/day, T, W	Undisturbed	P61		SPT, FST
Jaimes-Hoy et al., 2016 M	M	P2-P21, 3h/day, T, W	AFR	P40		EPM, OFT
Jaimes-Hoy et al., 2016 F	F	P2-P21, 3h/day, T, W	AFR	P40		EPM, OFT

Author	Sex	Format of Separation	Control	Age of First Test	Second Stress	Behavioural Test
Jaimes-Hoy et al., 2016 M	M	P2-P21, 3h/day, T, W	AFR	P60		EPM, OFT
Jaimes-Hoy et al., 2016	F	P2-P21, 3h/day, T, W	AFR	P60		EPM, OFT
Jin et al., 2018 M	M	P0-P20, 3h/day	Undisturbed	P49		EPM
Jin et al., 2018 F	F	P0-P20, 3h/day	Undisturbed	P49		EPM
Jones et al., 2009	M	P2-P14, 3h/day, I, W	MS15	P70		EPM, OFT
Kim et al., 2020	M	P2-P20, 3h/day, I	AFR	P35		FST
Lajud et al., 2012	M	P1-P14, 3h/day, T, W	AFR	P60		EPM, FST
J.-H. Lee et al., 2014	M	P1-P14, 3h/day, T, RT	AFR	P56		EPM, FST
Y. J. Lee et al., 2020	MF	P2-P14, 3h/day	AFR	P74		EPM
León Rodríguez and Dueñas, 2013 M	M	P1-P21, 3h 2X/day, T, W	AFR	P65		EPM
León Rodríguez and Dueñas, 2013 F	F	P1-P21, 3h 2X/day, T, W	AFR	P65		EPM
Li et al., 2013	M	P1-P21, 4h/day, I, W	Undisturbed	P35		EPM
Majcher-Maślanka et al., 2019	M	P2-P16, 3h/day, T, W	AFR	P35		SPT
Maniam and Morris, 2010b M	M	P2-P14, 3h/day, T, W	Undisturbed	P77		EPM
Maniam and Morris, 2010b F	F	P2-P14, 3h/day, T, W	Undisturbed	P77		EPM
Maniam and Morris, 2010a	M	P2-P14, 3h/day, T, W	MS15	P70		EPM, FST
Marais et al., 2008	M	P2-P14, 3h/day, T, W	Undisturbed	P65		FST
Marais et al., 2008 s	M	P2-P14, 3h/day, T, W	Undisturbed	P65	Chronic Restriant	FST
Melo et al., 2018	M	P2-P14, 3h/day, T, W	Undisturbed	P85		EPM, OFT
Oines et al., 2012	M	P2-P14, 3h/day, T, W	Undisturbed	P66		EPM
Papadakakis et al., 2019	M	P2-P14, 3h/day, T, RT	AFR	P81		EPM
Peng et al., 2019	M	P2-P14, 3h/day, T, W	AFR	P17		EPM, OFT
Ploj et al., 2002	M	P1-P21, 6h/day, T, RT	Undisturbed	P22		EPM
Ploj et al., 2002 s	M	P1-P21, 6h/day, T, RT	Undisturbed	P61	Isolation	EPM
S. Roque et al., 2014 E	M	P1-P14, 3h/day, T, W	Undisturbed	P90		OFT, FST
S. Roque et al., 2014 L	M	P1-P14, 6h/day, T, W	Undisturbed	P90		OFT, FST
A. Roque et al., 2020	M	P1-P14, 3h/day, T, W	AFR	P120		EPM, FST
A. Roque et al., 2021	M	P1-P14, 3h/day, T, W	AFR	P59		FST
Ruiz et al., 2018	M	P1-P14, 3h/day, T, W	AFR	P120		FST
Ruiz et al., 2018	M	P1-P14, 3h/day, T, W	AFR	P300		FST
Sadeghi et al., 2016	M	P2-P14, 3h/day	Undisturbed	P60		SPT, FST
Salberg et al., 2020 M	M	P2-P13, 4h/day, T, W	Undisturbed	P28		EPM
Salberg et al., 2020 F	F	P2-P13, 4h/day, T, W	Undisturbed	P28		EPM
Salberg et al., 2020 Ms	M	P2-P13, 4h/day, T, W	Undisturbed	P38	Sleep Deprivation	EPM
Salberg et al., 2020 Fs	F	P2-P13, 4h/day, T, W	Undisturbed	P38	Sleep Deprivation	EPM
Salzberg et al., 2007 M	M	P2-P14, 3h/day, I, W	MS15	P49		EPM

Author	Sex	Format of Separation	Control	Age of First Test	Second Stress	Behavioural Test
Salzberg et al., 2007 F	F	P2-P14, 3h/day, I, W	MS15	P49		EPM
Seo et al., 2016	M	P1-P21, 3h/day, T, W	AFR	P78		FST
Seo et al., 2016 s	M	P1-P21, 3h/day, T, W	AFR	P78	Chronic Restraint	FST
Shi et al., 2021	M	P4-P20, 4h/day, T	Undisturbed	P75		EPM, OFT
Shi et al., 2021 s	M	P4-P20, 4h/day, T	Undisturbed	P75	Chronic Restraint	EPM, OFT
Shu et al., 2015	M	P2-P14, 3h/day, T, RT	AFR	P91		SPT
Shu et al., 2015 s	M	P2-P14, 3h/day, T, RT	AFR	P124	VCS	SPT
Slotten et al., 2006 M	M	P3-P15, 3h/day, T, W	Undisturbed	P90		EPM
Slotten et al., 2006 F	F	P3-P15, 3h/day, T, W	Undisturbed	P90		EPM
Solarz et al., 2021	M	P1-P14, 3h/day, T, W	AFR	P70		SPT, FST
Stevenson et al., 2009	F	P2-P14, 6h/day, T, W	AFR	P90		OFT
Stuart et al., 2019	M	P1-P14, 3h/day, T, W	Undisturbed	P155		SPT
Trujillo et al., 2016	M	P1-P21, 4.5h/day, T	AFR	P74		EPM
Trujillo et al., 2016 s	M	P1-P21, 4.5h/day, T	AFR	P74	VCS	EPM
Tsotsokou et al., 2021	M	P1-P21, 3h/day, T, W	Undisturbed	P90		EPM, OFT
van Zyl et al., 2013	M	P2-P14, 3h/day, T, W	AFR	P72		FST
van Zyl et al., 2013 s	M	P2-P14, 3h/day, T, W	AFR	P72	Chronic Restraint	FST
Vargas et al., 2016	M	P1-P15, 3h/day, I, W	AFR	P58		FST
Wang et al., 2015 (21)	MF	P1-P21, 4h/day, I, W	Undisturbed	P21		OFT
Wang et al., 2015 (35)	MF	P1-P21, 4h/day, I, W	Undisturbed	P35		OFT
Wang et al., 2015 (56)	MF	P1-P21, 4h/day, I, W	Undisturbed	P56		OFT
Wei et al., 2018 M	M	P4-P10, 6h/day, T	AFR	P56		OFT, SPT, FST
Wei et al., 2018 F	F	P4-P10, 6h/day, T	AFR	P56		OFT, SPT, FST
Wei et al., 2018 Ms	M	P4-P10, 6h/day, T	AFR	P84	VCS	OFT, SPT, FST
Wei et al., 2018 Fs	F	P4-P10, 6h/day, T	AFR	P84	VCS	OFT, SPT, FST
Xiong et al., 2014	F	P2-P14, 3h/day, T	AFR	P70		EPM, OFT

Table 1 shows the key information extracted from each study. Where initials: T = litter separated together, I = individual pup separation, W = artifically warmth during separation, RT = room temperature during separation. For second stressors, VCS = variable chronic stress.
