# Supplementary material for: “The wrong tools for the right job”: a critical meta-analysis of traditional tests to assess behavioural impacts of maternal separation
Source: Psychopharmacology (Berl). 2022 Nov 23;240(11):2239–56. doi: 10.1007/s00213-022-06275-6 (PMC10593619; doi:10.1007/s00213-022-06275-6)
Supplement: Supplementary file 2 — Supplementary file2 (DOCX 181 KB) [file 213_2022_6275_MOESM2_ESM.docx]

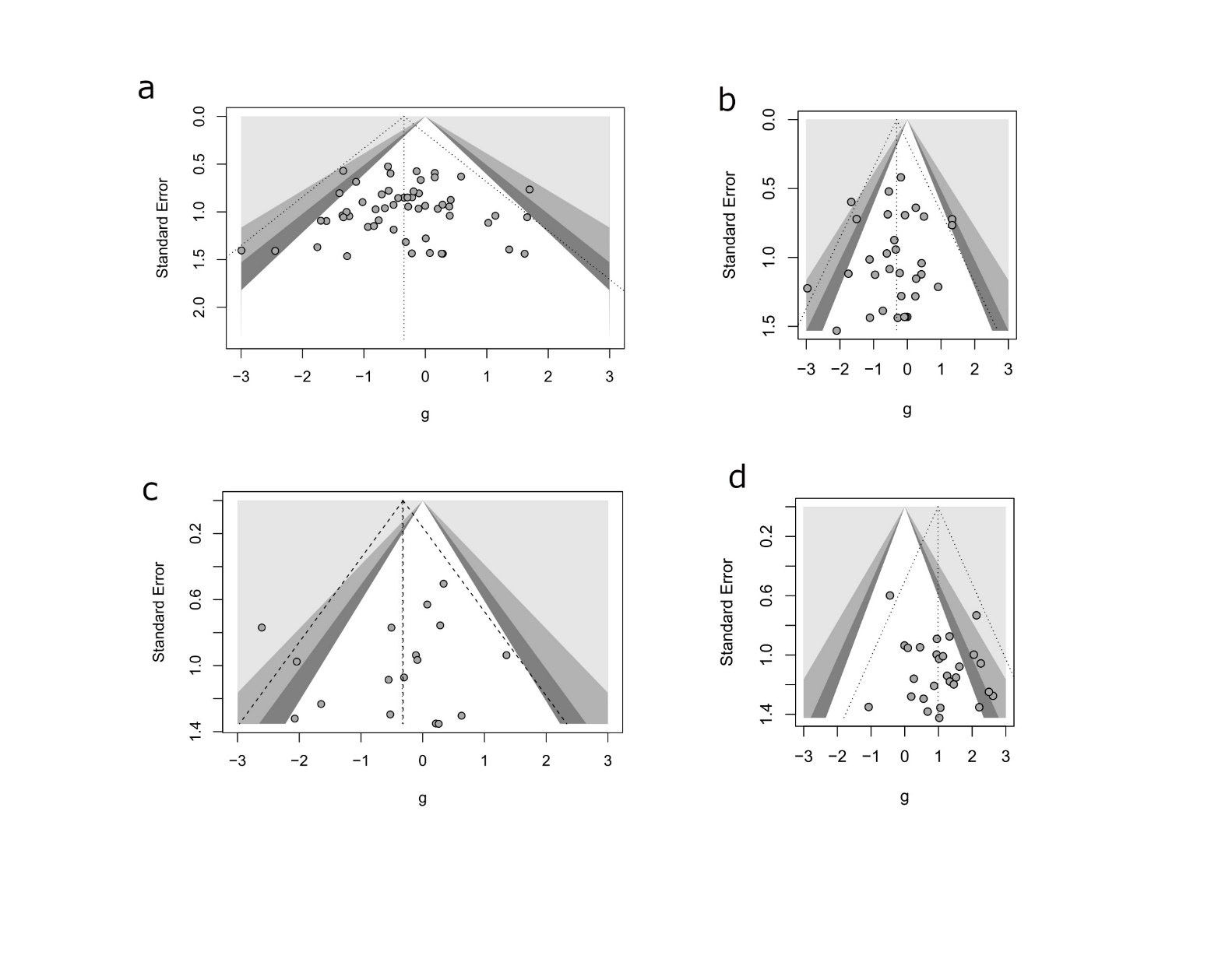


**Fig.S2.** Funnel plots for the different unconditioned behaviours discussed in the manuscript. Dotted lines show the distribution of reported effect sizes. Shaded regions indicate the significance of the reported effect size. (a) Elevated plus maze, (b) Open field test, (c) Sucrose preference test, (d) Forced swim test. The symmetry of each plot indicates that there was no publication bias in the studies selected for the meta-analyses.
